# Nitrogen Fixation Genes and Nitrogenase Activity of the Non-Heterocystous Cyanobacterium *Thermoleptolyngbya* sp. O-77

**DOI:** 10.1264/jsme2.ME17015

**Published:** 2017-11-23

**Authors:** Ki-Seok Yoon, Nga T. Nguyen, Kien Trung Tran, Kohsei Tsuji, Seiji Ogo

**Affiliations:** 1 International Institute for Carbon-Neutral Energy Research (WPI-I2CNER), Kyushu University 744 Moto-oka, Nishi-ku, Fukuoka 819–0395 Japan; 2 Center for Small Molecule Energy, Kyushu University 744 Moto-oka, Nishi-ku, Fukuoka 819–0395 Japan; 3 Department of Chemistry and Biochemistry, Graduate School of Engineering, Kyushu University 744 Moto-oka, Nishi-ku, Fukuoka 819–0395 Japan

**Keywords:** N_2_ fixation, *nif* genes, genome analysis, *Thermoleptolyngbya*, non-heterocystous cyanobacteria

## Abstract

Cyanobacteria are widely distributed in marine, aquatic, and terrestrial ecosystems, and play an important role in the global nitrogen cycle. In the present study, we examined the genome sequence of the thermophilic non-heterocystous N_2_-fixing cyanobacterium, *Thermoleptolyngbya* sp. O-77 (formerly known as *Leptolyngbya* sp. O-77) and characterized its nitrogenase activity. The genome of this cyanobacterial strain O-77 consists of a single chromosome containing a nitrogen fixation gene cluster. A phylogenetic analysis indicated that the NifH amino acid sequence from strain O-77 was clustered with those from a group of mesophilic species: the highest identity was found in *Leptolyngbya* sp. KIOST-1 (97.9% sequence identity). The nitrogenase activity of O-77 cells was dependent on illumination, whereas a high intensity of light of 40 μmol m^−2^ s^−1^ suppressed the effects of illumination.

Biological N_2_ fixation is the process by which atmospheric N_2_ gas is converted into useful ammonia. This reaction is catalyzed by the nitrogenase complex (EC 1.18.6.1), which consists of two metalloprotein components of the molybdenum-iron (MoFe) protein and iron (Fe) protein (5, 15). Due to their important role in the global N_2_ cycle, molecular and biochemical characterizations of nitrogen-fixing systems have been intensively performed (3–5, 13, 15, 25, 29, 44). Among N_2_-fixing microorganisms, cyanobacteria play a crucial role in maintaining the N_2_ cycle in global environments, resulting in a significant contribution to aquatic N_2_ fixation (17, 47, 48). Cyanobacteria exhibit remarkable diversity in their morphological structures and have been classified into heterocystous and non-heterocystous cyanobacteria (3). Heterocystous cyanobacteria such as *Anabaena* and *Nostoc* species have terminally differentiated cells called heterocysts, which are specialized for N_2_ fixation (14, 29, 30, 43). Heterocysts appear to lack the O_2_-evolving activity of photosystem II (PSII), while non-heterocystous cyanobacteria contain PSII in their cells (3, 24). Therefore, the N_2_-fixing system of non-heterocystous cyanobacteria markedly differs from that of heterocystous cyanobacteria (3, 4). Based on N fixation studies on various cyanobacteria (3, 11, 31, 44), our understanding of N_2_-fixing systems in non-heterocystous cyanobacteria has significantly advanced. Cyanobacterial N_2_ fixation appears to occur temporarily and is regulated by the circadian rhythms of their growth conditions (4, 6, 11, 21, 22, 33, 38, 46). Consequently, the expression of nitrogenase activity appears to be influenced by photon flux densities and O_2_ concentrations (3, 4, 22, 38, 46).

During the course of our studies on oxygenic photosynthesis, we isolated the thermophilic cyanobacterium strain O-77 from a hot spring in Japan (24). Strain O-77 grows well in temperatures ranging between 35 and 60°C, exhibiting thermophilic behavior with an optimal growth temperature of 55°C under aerobic conditions in nitrate-containing medium (24). Due to its thermophilic adaptation, strain O-77 was divided into a new genus of *Leptolyngbya*-like thermophilic cyanobacteria, named *Thermoleptolyngbya* sp. O-77 (*Tl.* O-77) (34). The new genus of *Thermoleptolyngbya* is distinct from that of *Leptolyngbya*. Although more than 100 genomes of cyanobacteria have been sequenced (20, 36, 37), the *nif* genes and N_2_-fixing systems from thermophilic cyanobacteria have not yet been examined in detail (1, 16, 23, 39–41). In the present study, we elucidated the genome sequence of *Tl.* O-77 and analyzed nitrogenase-encoding genes, together with its accessory genes. We also investigated the induction conditions of nitrogenase and characterized reaction conditions and thermal stability.

## Materials and Methods

### Cell culture and cultivation conditions

Cell cultures were performed in a modified DH+Fe medium with a 10-L bioreactor (Model MBF-1000ME; EYELA, Tokyo Rikakikai, Tokyo, Japan) as described previously (24). Cells were grown in an 8-L culture medium containing 20 mM sodium nitrate under agitation at 150 rpm. The culture was conducted at 45°C under the constant flow (200 mL min^−1^) of an aerobic gas mixture (dried air/CO_2_=99/1 [v/v%]) and constant illumination of 60 μmol m^−2^ s^−1^ using four 60 W incandescent lamps. This is the general cultivation method of *Tl.* O-77 to prepare a seed culture and collect cells for a genome analysis and biochemical studies on this strain.

### Genome sequencing

The Qiagen Genomic-tip 100/G (Valencia, California, USA) was used for DNA extraction. Approximately 10,000 μg of pure DNA was used for sequencing. Genome sequencing was performed using the PacBio RSII platform on single-molecule real-time (SMRT) cells with a 17-kb insert library (10). Reads were assembled *de novo* using the Hierarchical Genome Assembly Process version 3.0 (HGAP3) (7). Sickle version 1.2 was used for trimming the 3′-end and 5′-end when quality was low and high, respectively. In order to attain better accuracy, we conducted an assembly error correction using the iCORN2 (V0.95) program (26) repeated five times. The positions of protein-coding sequences, tRNA and rRNA genes were identified using the Prokka software tool for rapid annotation of the prokaryotic genome (35).

### Phylogenetic analysis

All NifH protein sequences were retrieved from the NCBI protein database. In the present study, we used the 54 selected full-length NifH sequences based on their high pairwise identity, along with the represented N_2_-fixing microorganisms. The NifH sequences were then aligned by MUSCLE (9) and a phylogenetic tree was reconstructed using MEGA version 6.0 (42) by applying the maximum likelihood method with 1,000 bootstrap replicates for values ≥50%. The LG substitution model with Gamma distribution and invariant site (LG+G+I) was used to model evolutionary rate differences among sites. In tree construction, all positions containing gaps and missing data were eliminated. The tree was drawn to scale, with branch lengths measured in the number of substitutions per site.

### Measurement of growth rates

The growth rates of cells were assessed by measuring the amount of increased chlorophyll (Chl) *a* during the cell culture. In order to ensure that Chl *a* of each sample was extracted, extractions were performed three times in absolute methanol. The resulting extract was centrifuged at 10,000×*g* at 4°C for 10 min. The Chl *a* concentration of the supernatant was assessed by Porra’s method (28).

### Nitrogenase induction in sealed vials

Cells cultured in nitrate-containing medium were washed twice with nitrate-free medium. The washed cells were used under all experimental conditions. Under nitrate-containing conditions, cells suspended again in nitrate-containing medium were grown under two different conditions: an aerobic gas mixture (dried air/CO_2_= 99/1 [v/v%]) and anaerobic gas mixture (Ar/N_2_/CO_2_=90/9/1 [v/v/v%]). Under nitrate-free conditions, cells suspended in nitrate-free medium were grown under five different N_2_ gas contents in inert gas mixtures: (N_2_/CO_2_=99/1 [v/v%]), (Ar/N_2_/CO_2_=69/30/1 [v/v/v%]), (Ar/N_2_/CO_2_=79/20/1 [v/v/v%]), (Ar/N_2_/CO_2_=90/9/1 [v/v/v%]), and (Ar/CO_2_=99/1 [v/v%]). The culture vials (total volume of 27 mL) containing 10 mL of cells were sealed with rubber stoppers and then flushed for 5 min with each of the different gas mixtures. Cell cultures were performed under the continuous illumination of 8 μmol m^−2^ s^−1^ using four 60 W incandescent lamps at 45°C. The five samples were collected and used to measure acetylene reduction in 12-h time intervals during the culture period of 72 h.

### Nitrogenase induction in a bioreactor

Cells cultured in the presence of 20 mM sodium nitrate were harvested and then washed twice with nitrate-free medium. Washed cells were transferred into a 10-L bioreactor containing 8 L of nitrogen compound-free medium. Cells were then cultured at 45°C under agitation at 150 rpm with the constant flow of the inert gas mixtures (Ar/N_2_/CO_2_=90/9/1 [v/v/v%], 120 mL min^−1^) under continuous illumination at 8 μmol m^−2^ s^−1^ using four 60 W incandescent lamps. Seventy milliliters of cells in the bioreactor were collected by a bioreactor sampling valve in 3-h time intervals and the 5 prepared vials, each containing 10 mL of cells, were used to assess nitrogenase activity.

### Nitrogenase assay

Nitrogenase assays were conducted in serum vials (total volume of 27 mL) containing 10 mL of the cell suspension. Sample vials were sealed and flushed with argon gas at room temperature for 5 min. The assay was initiated using an injection of 1 mL of acetylene gas (99.6%, gas cylinder of 5.0 kg) passed through an oil bubbler connected to a Schlenk line. The reaction was performed at 40°C for 1 h at 8 μmol m^−2^ s^−1^ illumination and then terminated by the addition of 0.3 mL of 30% trichloroacetic acid. Nitrogenase activity was routinely assessed based on the acetylene reduction reaction (8). Ethylene was measured by a gas chromatograph (7890B; Agilent Technologies, Santa Clara, CA, USA) with flame ionization detection (FID) fit with a GS-GASPRO capillary column (0.32 mm×30 m, Agilent J&W GC columns; Agilent Technologies). Helium was used as the carrier gas at a constant flow of 25 mL min^−1^; the temperatures of the oven, detection, and injection port were 40, 300, and 46.8°C, respectively. The amount of ethylene produced by each sample was calculated based on the standard curve obtained from different concentrations of ethylene gas (GL Sciences, Tokyo, Japan). The specific activity of nitrogenase was expressed as unit mg^−1^ of Chl *a*, where 1 unit is equal to the production of 1 nmol of ethylene h^−1^.

### Effects of light intensity on nitrogenase activity

The effects of light on nitrogenase activity were assessed under various light intensities. Cells cultured for 9 h in a bioreactor were used in this study. A cell suspension (10 mL) in 27-mL vials was flushed with Ar gas for 5 min. Each sample injected with 1 mL of acetylene gas was incubated at 40°C for 1 h under various photon flux densities (dark, 4, 8, 16, 24, and 40 μmol m^−2^ s^−1^) and the rate of acetylene reduction was measured. The desired light intensities were adjusted with an external voltage controller. The level of light intensity was assessed by a LX-1108 light meter (Lutron, Taipei, Taiwan).

### Effects of temperature on nitrogenase activity

The optimum temperature for nitrogenase activity was assessed by measuring acetylene reduction activity over a temperature range of 30–60°C. Each sample (10 mL) in 27-mL vials was flushed with Ar gas for 5 min, injected with 1 mL of acetylene gas, incubated at each temperature for 1 h under 8 μmol m^−2^ s^−1^ illumination, and the rate of acetylene reduction was then measured. The heat tolerance of nitrogenase activity was also evaluated by measuring retained activity after thermal incubation for 1 h under anaerobic conditions with Ar gas. Each sample, after an incubation over a temperature range of 30–60°C under 8 μmol m^−2^ s^−1^ illumination, was then subjected to an assay of residual nitrogenase activity. Under all assay conditions, cells cultured for 9 h in a bioreactor were used. Assay conditions were the same as those described in the method for the nitrogenase assay.

### Nucleotide sequence accession numbers

The complete genome sequences of *Tl.* O-77 in this study were deposited in the DDBJ/EMBL/GenBank database with the accession number of AP017367. Annotation and nucleotide sequence data are currently available to the public at the DDBJ/NCBI website.

## Results and Discussion

### Genome analysis of *Tl.* O-77

In the present study, we elucidated the whole genome sequence of *Tl.* O-77 and obtained a total of 70,723 reads with a read length of 598,195,020 bp. Total bases and read counts were 2,749,149,704, and 27,219,304, respectively. The complete genome of *Tl.* O-77 consists of a single chromosome with a length of 5,480,261 bp (Fig. S1). Prediction results showed that there were 4,865 potential protein-encoding genes, 45 tRNA genes, and six rRNA genes, with a GC content of 55.9% (Table S1, Fig. S1). The predicted protein-encoding genes were subjected to a similarity search between protein sequences against the non-redundant protein database using the BLASTP program (2). Among 4,865 potential protein-encoding genes, 1,786 sequences showed significant similarity to hypothetical proteins, accounting for 37%. Approximately 63% of the sequences showed high similarity to proteins with known functions from registered genes. In comparisons with the *Leptolyngbya* species, the genome sequence data of which are currently available in the database, the genome size and protein-encoding genes of *Tl.* O-77 were similar to those of *Leptolyngbya* sp. PCC 7376 and *Leptolyngbya* sp. PCC 6404, whereas its genome size was smaller than those of *Leptolyngbya* sp. Heron Island, *Leptolyngbya boyana* PCC 6306, and *Leptolyngbya* sp. KIOST-1 (19, 27, 36, 37). The chromosomal GC content of *Tl.* O-77 was markedly higher than those of most of the analogous *Leptolyngbya* species and, thus, may reflect its thermophilic behavior in living environments.

### Nitrogen fixation gene cluster and phylogenetic analysis

**Fig. 1:**
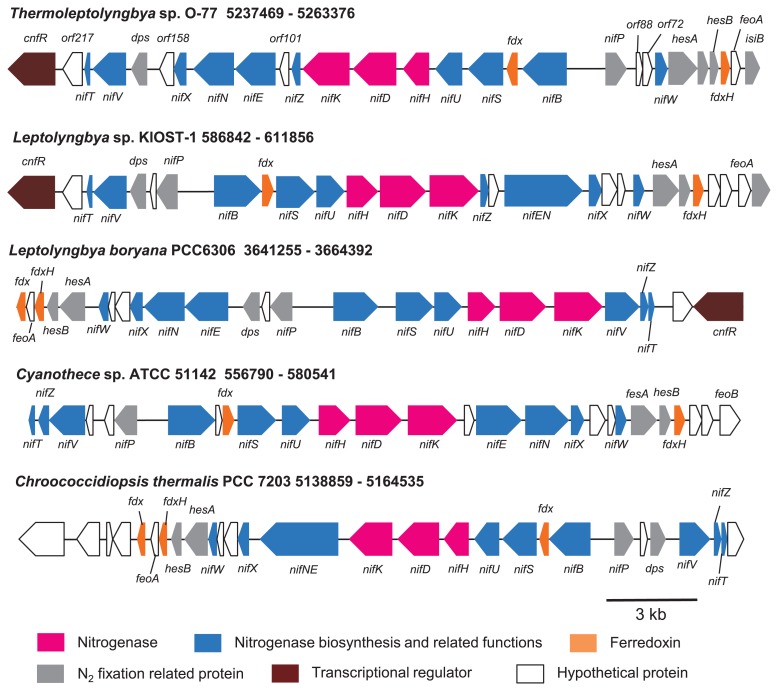
Nitrogen fixation gene cluster of *Thermoleptolyngbya* sp. O-77 and other non-heterocystous cyanobacteria. The arrow indicates the transcriptional direction (left/minus; right/plus).

The annotation result showed that *Tl.* O-77 contained at least 29 genes involved in nitrogen fixation (Fig. 1, Table S2). The *nif* genes of the FeMo cofactor synthesis and maturation components were identified from the genome sequence of *Tl.* O-77. Similar to other cyanobacterial N_2_ fixation genes (32), the *nif* gene cluster of *Tl.* O-77 is also divided into two regions with different transcriptional directions (Fig. 1). The *nifDKH* genes lie between 11 genes (*nifZ*, *orf101*, *nifE*, *nifN*, *nifX*, *orf158*, *dps*, *nifV*, *nifT*, *orf217*, and *cnfR*) and four genes (*nifB*, *fdx*, *nifS*, and *nifU*). The existence of *cnfR*, a transcriptional regulator, suggested that *Tl.* O-77 is responsible for anaerobic nitrogen fixation under low-oxygen conditions (44). The localization and direction of some genes of the N_2_-fixing gene cluster of *Tl*. O-77 were different from those of *L. boryana* PCC 6306 and *Cyanothece* sp. ATCC 51142, but were similar to those of *Leptolyngbya* sp. KIOST-1 and the thermophilic cyanobacterium *Chroococcidiopsis thermalis* PCC 7203; however, some genes such as *nifW*, *nifT*, and *nifV* were inversely localized.

We also constructed a phylogenetic tree of NifH (Fig. S2). The resulting phylogenetic tree showed that NifH sequences are classified into three groups. Groups I, II, and III are each composed of NifH from cyanobacteria, facultative anaerobes, and anaerobes, respectively. NifH of *Tl*. O-77 found in group I was placed in the cluster of *Leptolyngbya* species. All known *Leptolyngbya* species are mesophilic. The NifH sequence of *Tl*. O-77 was almost identical to that of *Leptolyngbya* sp. KIOST-1 (97.9% sequence identity), but distantly related to that of *L. boryana* PCC 6306 (87.9% sequence identity). Based on the N_2_-fixing gene cluster and NifH analysis, including phylogenetic re-classifications based on 16S rRNA gene sequences (34), we noted that the family of *Leptolyngbyaceae* was widely divergent across species. Our results provide important insights into the N_2_-fixing system of the thermophilic cyanobacterium *Tl*. O-77.

### Effects of the gas phase and nitrate on nitrogenase induction in sealed vials

In previous studies (12, 18, 45), the induction of nitrogenase activity in cells was performed under various N_2_ gas phases; however, the optimal N_2_ content has not yet been adequately examined. In order to establish optimal induction conditions for nitrogenase in *Tl*. O-77, we monitored nitrogenase activity in various N_2_ contents of inert gas mixtures with/without nitrate in a sealed vial system (Table 1). In nitrate-rich culture medium, *Tl*. O-77 cells did not exhibit nitrogenase activity. In contrast, in nitrate-free culture medium, *Tl.* O-77 cells exhibited strong nitrogenase activity. The strongest activity was observed in lower N_2_ content (Ar/N_2_/CO_2_=90/9/1 [v/v/v%]) rather than higher N_2_ gas phases (20, 30, 99 [v%]) and no N_2_ gas content (Ar, 99%). In comparison to other non-heterocystous cyanobacteria, the strong nitrogenase activity of *Plectonema boryanum* was also induced in culture conditions of a low N_2_ content of the gas mixtures (Ar/N_2_/CO_2_=72/24/4 [v/v/v%]) (18). In the case of heterocystous cyanobacteria such as *Anabaena variabilis* and *Nostoc spongiaeforme*, nitrogenase induction was stronger under the culture conditions of argon-based gas mixtures (Ar/CO_2_=99/1 [v/v%]) than N_2_-based gas mixtures (N_2_/CO_2_=99/1 [v/v%]) (45). In non-cyanobacteria, the nitrogenase activity of *Klebsiella pneumoniae* was weaker under the conditions of N_2_-based gas mixtures (N_2_/CO_2_= 99/1 [v/v%]) than argon-based gas mixtures (Ar/CO_2_= 99/1 [v/v%]) (12). These findings are consistent with our experimental results, suggesting that the N_2_ concentration in culture medium significantly affected the induction of nitrogenase activity by the thermophilic cyanobacterium of *Tl.* O-77.

### Time-course of nitrogenase activity

**Fig. 2:**
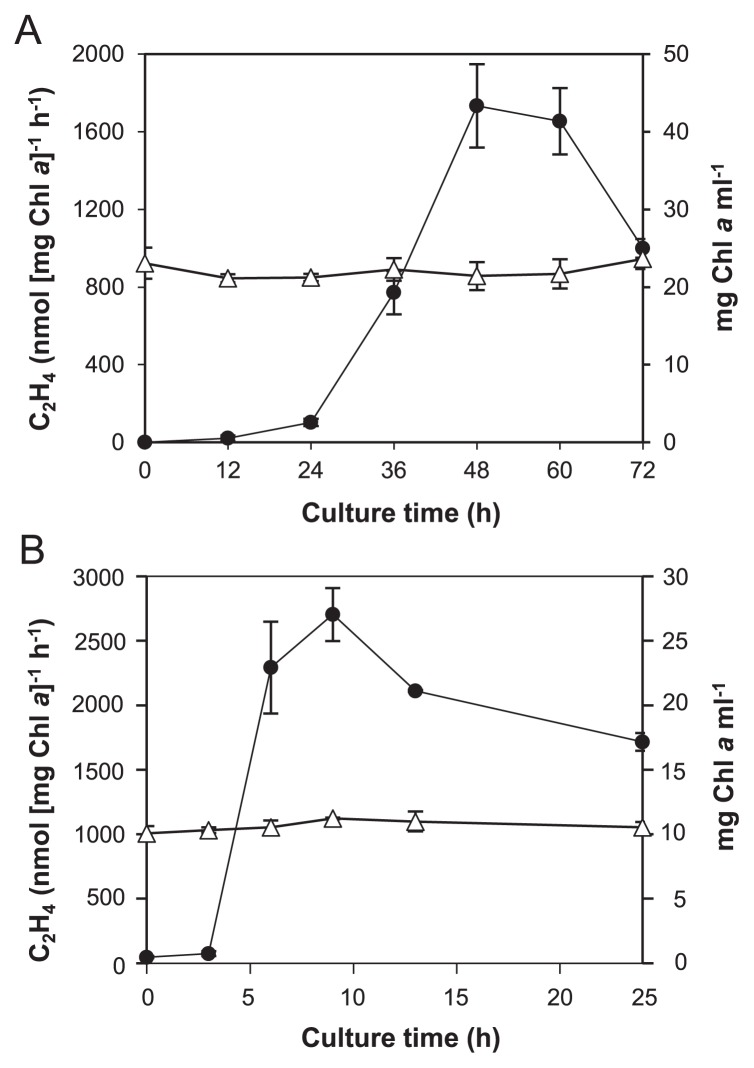
Comparisons of the nitrogenase activity and cell growth curve of *Tl*. O-77 under different culture conditions. (A) Induction of nitrogenase activity in *Tl*. O-77 cultured in the sealed vial system under the gas phase (Ar/N_2_/CO_2_=90/9/1, v/v/v%). The nitrogenase activity (●) and growth curves (△) of cells were periodically measured in a time course of 12-h intervals. (B) Induction of nitrogenase activity in *Tl*. O-77 cultured in a bioreactor under the continuous flow of the gas mixture (120 mL min^−1^, Ar/N_2_/CO_2_=90/9/1 [v/v/v%]). The nitrogenase activity (●) and growth curves (△) of cells were periodically measured in a time course of 3-h intervals. Cells cultured in nitrate-containing medium were washed twice with nitrate-free medium and then used for all experimental conditions. Cell cultures of the sealed vials and bioreactor were performed at the same temperature of 45°C and the same photon flux density of 8 μmol m^−2^ s^−1^. The growth curves of cells were assessed by measuring Chl *a* concentrations. Error bars indicate the standard deviation of the mean values. Data represent the means of at least three independent experiments.

In order to elucidate nitrogenase induction in *Tl.* O-77 in more detail, we performed a series of time-course experiments in sealed vials and a bioreactor. In sealed vials, we did not detect any significant nitrogenase activity by cells cultured for 24 h (Fig. 2A). The strongest activity was detected for cells cultured for more than 48 h. In the large-scale culture using a 10-L bioreactor, nitrogenase activity was detected in cells cultured for 3 h and the strongest activity was observed in cells cultured for 9 h. As shown in Figs. 2A and 2B, cells grew very poorly in an anaerobic culture in nitrate-free medium.

### Effects of light intensity on nitrogenase activity

**Fig. 3:**
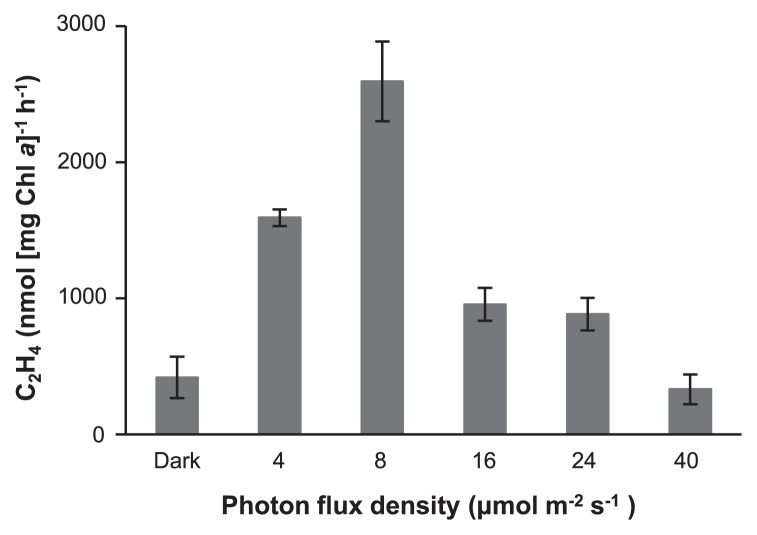
Effects of light intensity on the nitrogenase activity of *Tl*. O-77. Cells exhibiting strong nitrogenase activity (2704 nmol [mg Chl *a*] h^−1^) induced by using a bioreactor for 9 h were used for this experiment. All assays were performed at the same temperature of 40°C under the Ar gas phase. The desired photon flux densities between dark and 40 μmol m^−2^ s^−1^ were adjusted with an external voltage controller. Error bars represent the standard deviation of the mean values. Data represent the means of at least three independent experiments.

We investigated the effects of light intensity on nitrogenase activity by *Tl.* O-77 (Fig. 3). Cells cultured for 9 h in the bioreactor shown in Fig. 2B were used for this study. The experimental results suggest that the nitrogenase activity of *Tl.* O-77 is significantly dependent on light intensity. The strongest activity was detected at 8 μmol m^−2^ s^−1^, but activity was reduced at less than 4 μmol m^−2^ s^−1^. Photon flux density greater than 8 μmol m^−2^ s^−1^ significantly suppressed nitrogenase activity. Nitrogenase activity by *Tl.* O-77 appeared to be sensitive to a high light intensity. Based on these experimental results, high light intensity leads to an increase in the rate of the O_2_ evolution of PSII, resulting in the suppression of nitrogenase activity. Furthermore, low light intensity may not be sufficient to provide the substantial metabolic energy needed to drive N_2_ fixation (3). In natural environments, nitrogenase activity may be changed by the light-dark cycle in one d and *Tl*. O-77 may actively fix N_2_ at dawn and dusk. Previous studies reported that the gene expression of nitrogenase in non-heterocystous cyanobacteria was regulated by circadian rhythms (3, 6, 11, 21, 38). Further research is required to characterize the N_2_-fixing activity of *Tl*. O-77 in nature.

### Reaction temperature and heat tolerance of nitrogenase activity

**Fig. 4:**
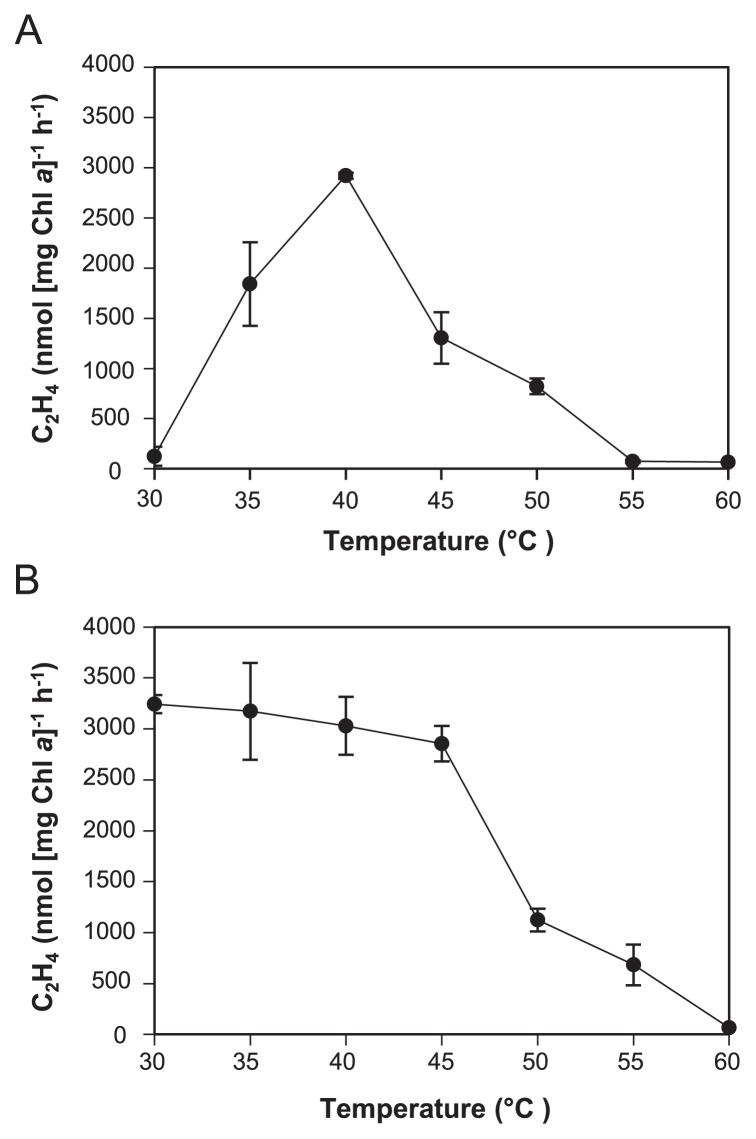
Optimal reaction temperature and heat tolerance of *Tl*. O-77 nitrogenase. (A) An optimal temperature between 30 and 60°C was assayed at 8 μmol m^−2^ s^−1^. (B) The heat tolerance of *Tl*. O-77 nitrogenase was examined in an incubation for 1 h between 30 and 60°C under the argon gas phase at 8 μmol m^−2^ s^−1^. Data were obtained from three independent experiments per condition. Error bars indicate the standard deviation of the mean values.

The effects of temperature on the nitrogenase activity of *Tl*. O-77 were investigated. The optimum temperature of nitrogenase activity was approximately 40°C, and activities detected at 45 and 50°C were similar at 45 and 28%, respectively (Fig. 4A). Nitrogenase activity was abolished at 55°C. According to this result, cell growth was severely impaired at 55°C in anaerobic nitrate-free medium (data not shown), whereas the optimum growth temperature of this strain was 55°C under aerobic conditions in nitrate-containing medium (24). The heat tolerance of nitrogenase activity was also examined by pre-incubating each sample under various temperatures between 30 and 60°C for 1 h. The nitrogenase activity of *Tl.* O-77 was stable up to 45°C and gradually decreased at higher temperatures (Fig. 4B). These results indicate that nitrogenase activity was sensitive to elevated temperatures such as 55°C, resulting in the failure to grow under N_2_-fixing conditions at high temperatures.

## Conclusion

We conducted a whole genome analysis on *Tl*. O-77, and the results obtained indicated that the genome consisted of a single chromosome with a length of 5,480,261 bp containing 4,865 potential protein-encoding genes. We assigned 29 *nif* genes from *Tl*. O-77 that were involved in the expression of a molybdenum-dependent nitrogenase complex. Based on the phylogenetic analysis, the NifH sequence of *Tl.* O-77 was the closest to that of *Leptolyngbya* sp. KIOST-1, but was distant to the formerly same genus of *L. boryana* PCC 6306. The nitrogenase activity of *Tl.* O-77 was highly dependent on light intensity and temperature. Further biochemical studies using the purified enzyme will provide more detailed insights into the N_2_-fixing system of thermophilic cyanobacteria.

## Supplementary Material



## Figures and Tables

**Table 1:** Effects of the gas phase and culture medium for the induction of nitrogenase activity in *Tl.* O-77

Gas phase and media (Percentage of gas partial pressure [v/v%])	Acetylene reduction (nmol [mg Chl *a*]^−1^ h^−1^)
Air/CO_2_ (99/1)_NaNO_3_ (20 mM)	N.D.
Ar/N_2_/CO_2_ (90/9/1)_NaNO_3_ (20 mM)	N.D.
N_2_/CO_2_ (99/1)_N-free medium	272±71
Ar/N_2_/CO_2_ (69/30/1)_N-free medium	712±8.2
Ar/N_2_/CO_2_ (79/20/1)_N-free medium	962±52
Ar/N_2_/CO_2_ (90/9/1)_N-free medium	1206±158
Ar/CO_2_ (99/1)_N-free medium	927±77

Cell cultures were performed in sealed vials (total volume of 27 mL) under different gas phases with a light intensity of 8 μmol m^−2^ s^−1^ at 45°C. Cells cultured in nitrate-containing medium were washed twice with nitrate-free medium and then used for all experimental conditions. After a culture for 48 h, the rate of acetylene reduction of each sample was measured. Data represent the means of at least three independent experiments. N.D.: not detected.
