# Supplementary material for: Nitrogen Fixation Genes and Nitrogenase Activity of the Non-Heterocystous Cyanobacterium Thermoleptolyngbya sp. O-77
Source: Microbes Environ. 2017 Nov 23;32(4):324–9. doi: 10.1264/jsme2.ME17015 (PMC5745016; doi:10.1264/jsme2.ME17015)
Supplement: Supplementary file 1 [file 32_324_s1.pdf]

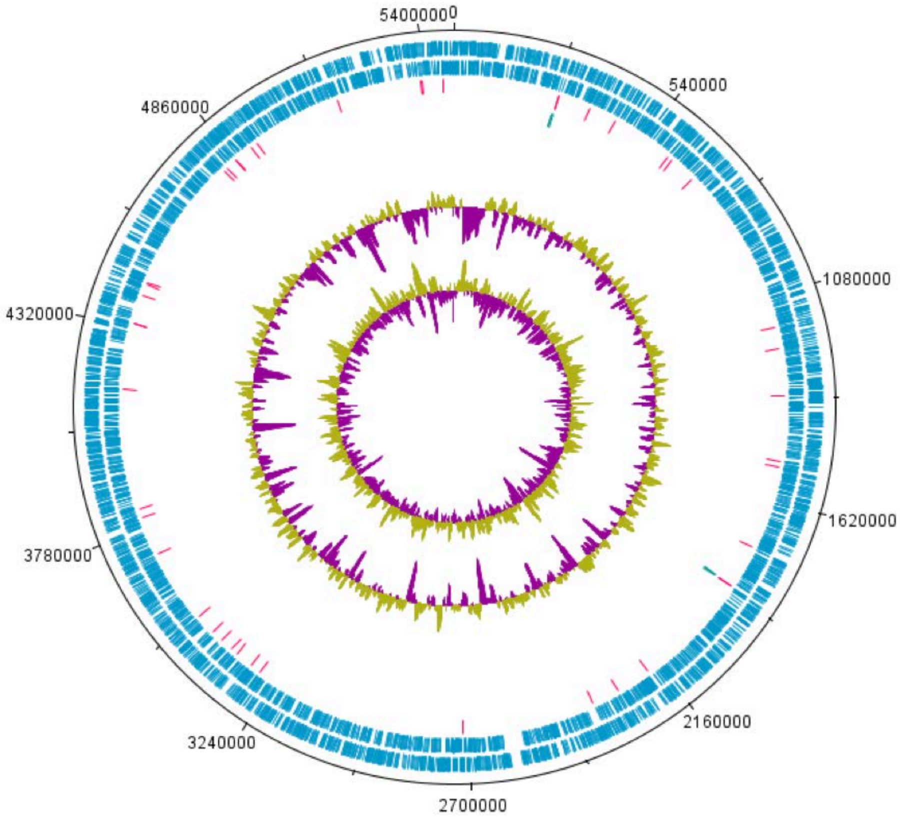

Fig. S1. Circular representation of the chromosome of *Thermoleptolyngbya* sp. O-77. The bars in the first and second circle indicate the putative protein of CSD on forward strand and reverse strand, respectively. The bar in the third circle shows tRNA genes and the fourth is rRNA genes. The fourth inner bar shows the GC content and the innermost circle shows the GC skew.

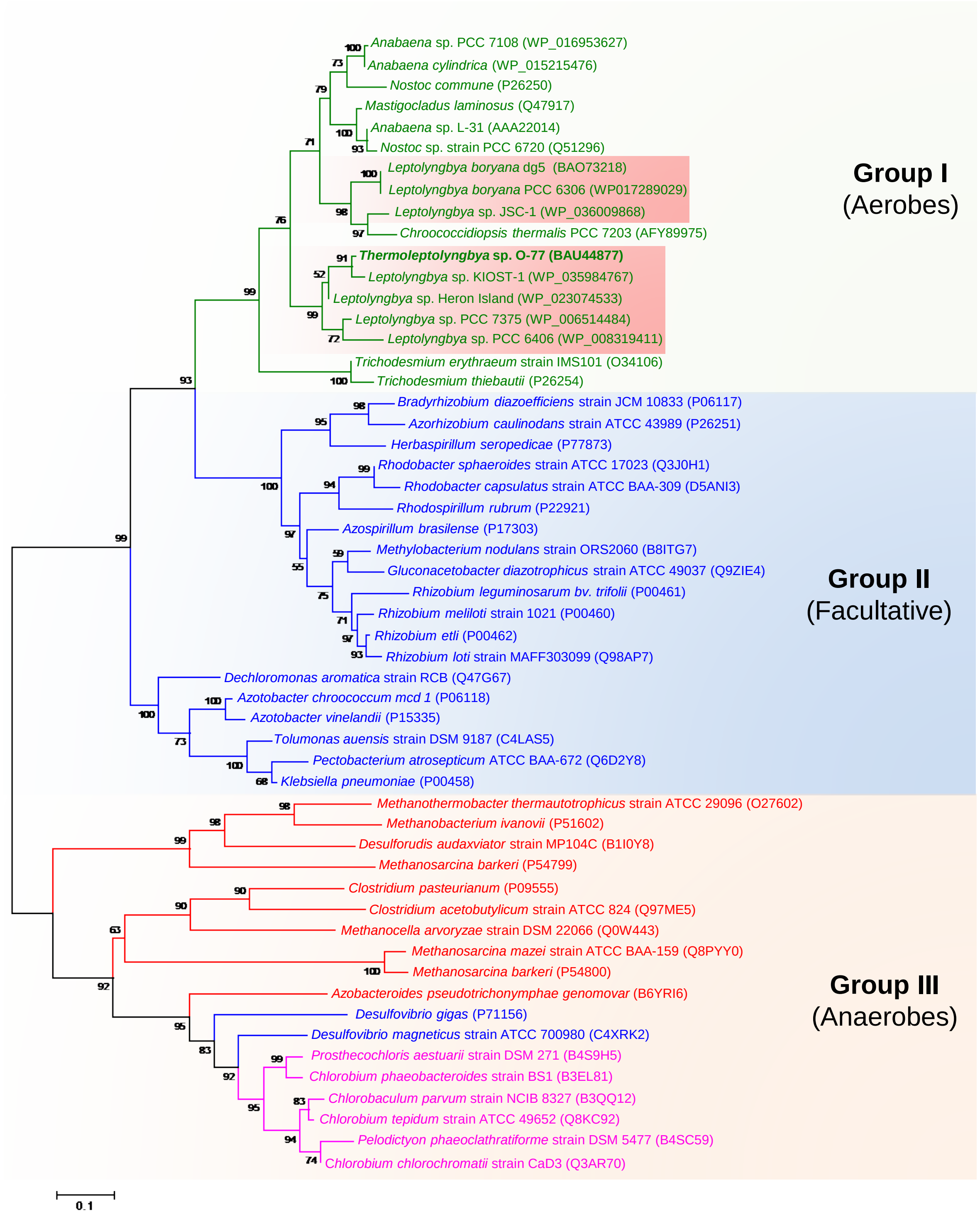

Fig. S2. Molecular Phylogenetic analysis of NifH by maximum likelihood method with 1000 times bootstrap replicates for values ≥50%. The percentage of trees in which the associated taxa clustered together is shown next to the branches. The scale bar represents the number of substitutions per site. The tree was classified into three groups based on the microorganism's living environment: Group I (aerobic); group II (facultative); group III (anaerobic). *Tl.* O-77 was shown in bold. Line and strains color represent the different taxa: cyanobacteria (green); proteobacteria (red); eukaryotes and Firmicutes (navy); green sulfur bacteria (pink). Two phylogenetic distinct types of *Leptolyngbya* species are highlighted in pink background. The tree was drawn to scale, with branch lengths measured in the number of substitutions per site.

Table S1. Comparative genome features

Species	*Tl.* sp. O-77	*L.* sp. Heron Island	*L. boryana* PCC 6306	*L.* sp. KIOST-1
GeneBank accession number	AP017367	AWNH00000000	ALVM00000000	JQFA00000000
Genome size (Mb)	5.48	8.06	7.26	6.32
Protein encoding genes (CDS)	4,865	6,690	6,524	5,327
GC content (%)	55.9	48	47	59.4
tRNA genes	45	54	67	45
rRNA genes	6	3	9	7
References	This study	Paul et al. (2014)	Shih et al. (2013)	Kim et al (2016)

Table S2. List of nitrogen fixation genes of *Thermoleptolyngbya* sp. O-77

Gene name	Length (AA)	Description	Accession number
cnfR	532	XRE family transcriptional regulator	WP_068515247
orf217	217	Hypothetical protein	WP_068515249
nifT	67	Nitrogen fixation protein NifT	WP_068515251
nifV	373	Homocitrate synthase NifV	WP_068515253
dps	180	DNA-binding ferritin-like protein	WP_068515255
orf158	158	Hypothetical protein	WP_068515272
nifX	141	Nitrogenase MoFe cofactor biosynthesis protein NifX	WP_068515278
nifN	456	FeMo cofactor biosynthesis	WP_068515280
nifE	491	FeMo cofactor biosynthesis protein NifE	BAU44872
orf101	101	Hypothetical protein	WP_068515281
nifZ	100	Nitrogenase biosynthesis	WP_068515283
nifK	525	Mo-Fe protein beta chain	WP_068515285
nifD	484	Nitrogenase. Mo-Fe protein alpha chain	WP_068515287
nifH	291	Nitrogenase Fe protein	WP_068515290
nifU	301	Formation of Fe-S clusters in iron-sulfur proteins	WP_068515292
nifS	397	Cysteine desulfurase NifS	WP_068515294
fdx	124	Produce [4Fe-4S] ferredoxin	WP_068515297
nifB	496	Synthesis of Fe-Mo cofactor	WP_068515299
nifP	241	Serine O-acetyltransferase	WP_068515300
orf88	88	Hypothetical protein	BAU44883
orf72	72	Hypothetical protein	WP_068515301
nifW	105	Nitrogenase stabilizing/protective protein	WP_068515304
hesA	260	Protein HesA	WP_068515314
hesB	122	Protein HesB	WP_068516892
fdxH	100	[2Fe-2S] ferredoxin	WP_068516893
feoA	103	Iron transporter	WP_068515317
isiB	176	Flavodoxin	WP_068515319
orf137	137	Hypothetical protein	WP_068515322
orf211	211	SAM-dependent Methyltransferase	WP_068516894

BLAST search against the redundant protein was conducted on 25 August 2016 (https://blast.ncbi.nlm.nih.gov/Blast.cgi).
